# Correction: Synovial Fluid Response to Extensional Flow: Effects of Dilution and Intermolecular Interactions

**DOI:** 10.1371/journal.pone.0106158

**Published:** 2014-08-13

**Authors:** The PLOS ONE Staff

## Notice of Republication

This article was republished on June 19, 2014, to correct errors in symbols that were introduced during the typesetting process. Please download this article again to view the correct version. The originally published, uncorrected article and the republished, corrected article are provided here for reference.

## Supporting Information

File S1 Originally published, uncorrected article.
(PDF)

File S2 Republished, corrected article.
(PDF)
