# Supplementary material for: Correction: Synovial Fluid Response to Extensional Flow: Effects of Dilution and Intermolecular Interactions
Source: PLoS One. 2014 Aug 13;9(8):e106158. doi: 10.1371/journal.pone.0106158 (PMC4132115; doi:10.1371/journal.pone.0106158)
Supplement: File S2 — Republished, corrected article. (PDF) [file pone.0106158.s002.pdf]

Synovial Fluid Response to Extensional Flow: Effects of Dilution and Intermolecular Interactions

Simon J. Haward*

Faculdade de Engenharia da Universidade do Porto, Departamento de Engenharia Química, Centro de Estudos de Fenómenos de Transporte, Porto, Portugal

Abstract

In this study, a microfluidic cross-slot device is used to examine the extensional flow response of diluted porcine synovial fluid (PSF) samples using flow-induced birefringence (FIB) measurements. The PSF sample is diluted to 10× 20× and 30× its original mass in a phosphate-buffered saline and its FIB response measured as a function of the strain rate at the stagnation point of the cross-slots. Equivalent experiments are also carried out using trypsin-treated PSF (t-PSF) in which the protein content is digested away using an enzyme. The results show that, at the synovial fluid concentrations tested, the protein content plays a negligible role in either the fluid's bulk shear or extensional flow behaviour. This helps support the validity of the analysis of synovial fluid HA content, either by microfluidic or by other techniques where the synovial fluid is first diluted, and suggests that the HA and protein content in synovial fluid must be higher than a certain minimum threshold concentration before HA-protein or protein-protein interactions become significant. However a systematic shift in the FIB response as the PSF and t-PSF samples are progressively diluted indicates that HA-HA interactions remain significant at the concentrations tested. These interactions influence FIB-derived macromolecular parameters such as the relaxation time and the molecular weight distribution and therefore must be minimized for the best validity of this method as an analytical technique, in which non-interaction between molecules is assumed.

Citation: Haward SJ (2014) Synovial Fluid Response to Extensional Flow: Effects of Dilution and Intermolecular Interactions. PLoS ONE 9(3): e92867. doi:10.1371/journal.pone.0092867

Editor: Tom Waigh, University of Manchester, United Kingdom

Received November 4, 2013; **Accepted** February 26, 2014; **Published** March 20, 2014

Copyright: © 2014 Simon J. Haward. This is an open-access article distributed under the terms of the Creative Commons Attribution License, which permits unrestricted use, distribution, and reproduction in any medium, provided the original author and source are credited.

Funding: This research was supported by a Marie Curie International Incoming Fellowship within the 7th European Community Framework Programme, FP7-PEOPLE-2011-IIF, grant 298220. The funders had no role in study design, data collection and analysis, decision to publish, or preparation of the manuscript.

Competing Interests: The author has declared that no competing interests exist.

* Email: shaward@fe.up.pt

Introduction

Synovial fluid (SF) plays a vital role in the protection and maintenance of healthy function in animal joints. In particular, the rheology of SF, which is highly shear-thinning and viscoelastic [1–3], contributes to the low friction between joint surfaces undergoing flexion. Extensional rheological measurements have also shown that SF is significantly strain-hardening under elongational deformations [3], which is likely to provide shock-dampening when joints experience sudden compressive loading, as occurs in the knees during running and jumping for example [4,5].

Synovial fluid is predominantly a semi-dilute solution of the glycosaminoglycan hyaluronic acid (HA), a high molecular weight linear polysaccharide. In healthy SF the HA concentration is around 0.3 wt.% and has a molecular weight of a few million Daltons [6]. Synovial fluid is an ultrafiltrate of blood and, as well as HA, also contains a significant amount of the serum proteins globulin (≈ 0.7 wt.%) and albumin (≈ 1.1 wt.%) [7]. These proteins may interact with the HA macromolecules in SF and some authors have argued that such interactions can have important functional consequences on the SF rheology. For example, Oates et al. [8,9] compared the steady shear rheology of a pure HA solution with that of a model SF composed of HA plus bovine serum albumin and γ-globulins. In a concentric cylinder geometry, they observed phenomena including an enhanced viscosity of the HA-protein mixture at low shear rates ($\leq 1\ s^{-1}$) and rheopexy (a shear stress that increases over time) also at low shear rates, which they attributed to the formation of protein aggregates that entangle with the HA. However, more recently Sharma et al. [10] have shown that very similar effects can be observed in the rheometer due to the high surface activity of serum proteins and the resulting formation of a cohesive layer at the air interface of the rheometer geometry. In a 60 mm diameter 1° cone-and-plate geometry, Bingöl et al. [3] found negligible difference between either the shear or extensional rheological properties of pure HA solutions and models of synovial fluid. Furthermore, Bingöl et al. [3] found that the rheology of native human synovial fluid was very well approximated by a solution of 0.3 wt.% of high molecular weight HA ($M_W = 4.6$ MDa) with no added protein and they concluded that the SF rheological properties could be almost exclusively attributed to the HA alone. Recently, Haward et al. [5] performed rheological experiments with similar HA solutions and models of SF. In steady shear experiments using a 40 mm diameter 2° cone-and-plate geometry they observed the same kind of viscosity enhancements at low shear rates in the model SF that were previously reported by Oates et al. [8,9], but not by Bingöl et al. [3]. However, in the interface-free extensional flow experiment in a cross-slot device Haward et al. [5] found no observable difference between the response of the model SF and the pure HA solution. The fact that the observation of the apparent structure-building by the proteins in steady shear (as reported by Oates et al.) seems to depend upon the experimental flow geometry strongly hints at an interfacial as opposed to a bulk rheological effect [5,10]. It is crucial in terms of our understanding of the role of synovial fluid rheology in relation

to its physiological function that the uncertainty over the nature of serum protein interactions in SF be definitively resolved.

Another important protein that is present in the synovial fluid but has received relatively little attention from rheologists is lubricin. This probably has to do with the minute quantities of lubricin that are available, combined with its prohibitive cost. Lubricin is a mucinous glycoprotein that exists in SF at a concentration of $\approx$ 0.2 wt.% [11] and its primary function is generally regarded as providing boundary lubrication of cartilage surfaces under load. However, Jay et al. [12] have also shown evidence that lubricin may influence the bulk rheology of SF through interactions with the HA. In steady shear experiments they found that the viscosity of an HA solution decreased considerably after the addition of lubricin, and that the viscosity of a bovine SF sample increased after the protein had been digested by the enzyme trypsin. This would certainly seem not to be explained by protein aggregation at the air interface of the rheometer, which would be expected to cause an apparent increase in the fluid viscosity. In combination with measurements of the diffusive behaviour of tracer particles suspended in the test fluids, the rheological results of Jay et al. [12] suggest the HA adopts a more coiled and less extended conformation in the presence of lubricin. Without speculating on the nature of the interaction, they suggest that the lubricin renders the HA more deformable and thus facilitates greater energy dissipation over the loading cycles that occur within the joints during mammalian locomotion.

In a recent paper, Haward [13] performed a detailed study of the extensional response of dilute HA solutions over a range of molecular weights, 0.9 MDa $\leq M_W \leq$ 4.8 MDa and concentrations, 0.01 wt.% $\leq c \leq$ 0.07 wt.%. Flow-induced birefringence (FIB) measurements were made as a function of the strain rate, $\dot{\varepsilon}$, imposed at the stagnation point of a microfluidic cross-slot device. In such strong extensional flows, at beyond a critical strain rate ($\dot{\varepsilon}_c \approx 0.5/\tau$, where τ is the longest macromolecular relaxation time) long chain coiled molecules like HA in solution will undergo a coil-to-stretch transition, orienting and aligning in the direction of flow and leading to optical anisotropy, or FIB [14,15]. Such measurements of FIB in the cross-slots were used to establish a relationship $\tau \sim M^{1.8}$, consistent with expectations that HA is an expanded, semi-flexible partially solvated random coil at equilibrium [13,16]. In addition, it was found that the plateau value of the birefringence measured at high strain rates correlated linearly with the concentration of the HA in solution [13]. It was demonstrated how such FIB measurements within a microfluidic extensional flow device could be of potential use for the rapid assessment of the HA molecular weight and concentration in a small sample of diluted synovial fluid. However, the validity of such measurements depends upon the assumption of the diluteness of the HA macromolecules and that they are unperturbed from their natural conformation in the solvent. Real synovial fluid is clearly not a simple dilute aqueous solution of HA and contains a number of additional components of arguable rheological significance. Therefore, for the valid application of potential flow techniques to assess the HA parameters in real SF it is vital to ascertain how dilute the SF should be before the ideal behaviour of the HA macromolecules is achieved, and also whether any proteinaceous constituents in the SF influence that behaviour. The effects of potential HA-HA and protein-HA interactions in the synovial fluid was not investigated in the original study [13], and is the primary purpose of the current investigation. Intermolecular HA interactions in the SF sample will affect the macromolecular relaxation time, and hence the molecular weight determined from the measurement; the same will be true if there are protein-HA interactions in the sample that may affect the HA flexibility or coil dimensions, or indeed if protein-protein interactions affect the rheology of the surrounding medium in which the HA is present. The extensional flow technique is extremely sensitive to variations in macromolecular parameters, as has been demonstrated by experiments involving solutions of nearly monodisperse polymers in solvents of various viscosity and quality, and of polyelectrolytes in the presence of various concentrations of counterions [17]. Therefore the technique represents a good potential opportunity to learn more about the dynamical behaviour of the HA in synovial fluid.

In this article the influence of dilution upon the shear and extensional rheological response of synovial fluid samples is investigated using SF harvested from healthy porcine tarsal joint. In addition, to investigate the influence of protein-HA interactions, SF samples are treated with the trypsin enzyme in order to digest the protein content while leaving the HA macromolecules intact. This approach to investigating protein-HA interactions in the SF has the significant advantage over comparing pure HA solutions with models of synovial fluid (as in the works of Bingöl et al. [3], Haward et al. [5] and Oates et al. [8,9]) in that the undigested SF contains all the serum proteins and the lubricin, all of which are potentially implicated in the fluid rheology. The results of the study will be of significance to the validity of synovial fluid HA analysis, either by microfluidic or by other techniques where the SF is first diluted (for example GPC), and will also provide insight into the nature of intermolecular interactions between the various proteins and HA components of synovial fluid.

Materials and Methods

2.1 Ethics statement

The animal material used was purchased from a local butcher and was not produced for the purpose of the investigation. It is a by-product of the meat industry, available for consumption from some butchers, or otherwise part of the waste stream.

2.2 Test fluid preparation

Slightly more than 2 g of porcine synovial fluid (PSF) was harvested from the tarsal (ankle) joints of a freshly slaughtered healthy pig that had shown no sign of joint pathology prior to sacrifice. The PSF was centrifuged at 4000 g for 10 minutes to remove the bulk of extraneous material, divided into two equal quantities of approximately 1 g each and stored at -30°C in sterile Eppendorf containers until required for use. One quantity of the pure PSF was subdivided into three smaller volumes and diluted with a phosphate-buffered saline (PBS, 0.01 M, pH 7.4, Sigma Aldrich) to yield three quantities of between 5 g and 6 g of fluid at 10x, 20x and 30x their original undiluted mass (dilution factor f = 10, 20 and 30, respectively). This provided sufficient fluid at each dilution to allow duplicate testing in each subsequent rheological experiment. Prior to experimentation the diluted PSF samples were centrifuged for a second time at 4000 g for 10 minutes to further remove impurities.

To investigate the role of synovial fluid serum proteins and lubricin on the PSF rheological response, the second stored quantity of the pure PSF was digested with 1-tosylamide-2-phenylethyl chloromethyl ketone (TPCK)-treated trypsin (Isogen Life Sciences). A 25 µL aliquot of a 0.2 wt.% solution of trypsin in PBS was added to the $\approx$ 1 g of PSF and incubated for more than 2 hours at 37°C with intermittent gentle agitation. The trypsin enzyme digests the synovial fluid protein component without affecting the integrity of the HA macromolecules. Subsequent to incubation, the trypsin-treated PSF (which will from now on be

referred to as t-PSF) was subdivided into three smaller quantities and diluted in PBS by the same factors, *f*, as the undigested PSF. As with the diluted PSF samples, prior to rheological testing the t-PSF samples were also centrifuged again at 4000 *g* for 10 minutes.

To prevent any possibility of bacterial infestation of the PSF and t-PSF samples, 0.02 wt.% sodium azide (Merck) was also added to each sample.

2.3 Steady shear rheological measurements

Fluids were tested in steady shear at 25°C using an Anton Paar Physica MCR 301 stress controlled rheometer with a 75 mm diameter 1° cone-and-plate fixture. To prevent sample evaporation during the tests, a solvent trap and covering hood were utilized.

2.4 Extensional flow experiments

The response of the test fluids to an extensional flow field was examined using a microfluidic cross-slot device. Cross-slots consist of orthogonally bisecting rectangular channels. Fluid is pumped into two opposing channels and is forced to exit through the remaining two diametrically-opposed channels. Symmetry of the flow field results in a stagnation point at the centre of the device and planar extension of fluid elements flowing along the streamline directed outwards from the stagnation point along the centreline of the two exit channels. The cross-slots used in the present experiment have a channel width of $w = 200$ μm and a depth of $d = 1.05$ mm. The same device has been described in a few previous publications [18,19] and details of their construction and application to extensional rheometry with Newtonian and model viscoelastic fluids can be found therein. The nominal rate of extension, or strain rate in the device is given by:

$$\dot{\varepsilon} \approx \frac{1.7U}{0.5w} \quad (1)$$

where U is the average flow velocity in each channel and the numerator, $1.7U$, is the maximum flow velocity on the channel centreline. Flow velocimetry measurements have shown that Eq. (1) provides a good estimate of the strain rate at the stagnation point for Newtonian fluids and it should also be valid for weakly elastic fluids that do not significantly perturb the flow field [19]. The flow velocity, and hence $\dot{\varepsilon}$, is controlled by varying the amplitude and period of a triangular voltage signal applied across a system of oscillating piezoelectric micropumps. This method provides for extremely accurate and smooth displacements of very low fluid volumes. The system as a whole is known as the extensional flow oscillatory rheometer (EFOR), and has been described in detail several times previously [13,18,20].

Various dimensionless parameters are used to characterize the flow of complex fluids in the EFOR device. Since the flow is oscillatory in nature, both a Deborah number and a Weissenberg number are necessary to describe elastic effects. The Weissenberg number characterizes the strength of the extensional flow field at the stagnation point and is defined by:

$$\mathrm{Wi} = \dot{\varepsilon}\tau \quad (2)$$

where τ is the longest characteristic macromolecular relaxation time. The values of τ for the various SF-based test fluids will be determined directly from the results of the subsequent flow experiments (as described later on) and are presented in Table 1. Elastic effects associated with the onset of macromolecular deformations in the flow are expected to occur when $\dot{\varepsilon}$ exceeds a critical value such that Wi exceeds the value 0.5. The value of the Wi probed in the current experiments varies between approximately $0 < \mathrm{Wi} < 10$.

Table 1. Characteristic relaxation times of PSF and t-PSF samples at various dilution factors, determined from stagnation point birefringence measurements in a cross-slot device.

	**Characteristic relaxation time, τ [ms]**		
**Fluid sample**	**10× dilution**	**20× dilution**	**30× dilution**
PSF	5.0±0.3	4.2±0.3	2.8±0.2
t-PSF	5.2±0.4	5.0±0.3	3.0±0.2

doi:10.1371/journal.pone.0092867.t001

The Deborah number characterizes the ability of macromolecules to relax on the timescale of the imposed flow and, for flow in the EFOR, can therefore be defined as follows:

$$\mathrm{De} = 2\tau/\mathrm{T} \quad (3)$$

where T is the periodic cycle time of the micropumps driving the flow. In the experiments described here, the shortest value used for T is approximately 4 seconds, which results in the largest experimental value for the Deborah number (i.e. at the highest imposed strain rates) of $\mathrm{De} \approx 2\times10^{-3}$. This low value for De indicates that the macromolecules have ample time to relax given the timescale of the flow.

Inertial effects within the cross-slot microchannel are quantified by the magnitude of the Reynolds number, defined by:

$$\mathrm{Re} = \rho U D_h/\eta \quad (4)$$

where $\rho \approx 1000$ kg m^{-3} is the fluid density, $D_h = 2wd/(w+d) =$ 336 μm is the hydraulic diameter and η is the fluid viscosity, which is measured using standard rotational rheometry (see Section 3.1). Despite the relatively low viscosities of the test fluids employed, the small size scale of the cross-slot device used in the study means that the Reynolds numbers remain moderate. The maximum Reynolds number values at the highest imposed strain rates are $\mathrm{Re} \approx 20$.

2.5 Birefringence measurement

An optical line centred as closely as possible on the stagnation point is used to quantify the flow-induced optical anisotropy (FIB) that results from the orientation and alignment of the fluid microstructure as the strain rate is varied. The active components of the optical train consist of a temperature stabilized solid state laser (650 nm, 60 mW, OzOptics), polarizer, analyser, a λ/4-plate and a cooled 14-bit CCD camera (Andor Technology). The polarizer and analyser are positioned on opposite sides of the cross-slots and crossed at ±45° to the direction of flow. The λ/4-plate is inserted and rotated until the transmitted intensity from the laser is minimized, thus compensating for any residual birefringence in the system. Subsequently, the polarizer and analyser are adjusted iteratively to achieve the maximum extinction. Finally, the polarizer is uncrossed slightly (by <3°) allowing a background signal to be detected by the CCD. This technique has the advantages (over simply using crossed polarizers, for example) of increasing the signal to noise ratio and also linearizing the usually quadratic relationship between the

birefringence, Δn, and the light intensity measured at the CCD [21]. The measured intensity as a function of the strain rate is calibrated to a *retardation*, R, by using a rotatable Brace-Köhler $\lambda/30$ compensator to introduce known values of R into the optical line. The relationship between retardation and birefringence is given by $R = d\Delta n$, where d is the pathlength through the birefringent material, which is taken to be equal to the depth of the flow cell.

For each imposed value of $\dot{\varepsilon}$, a video is captured with the image acquisition settings (i.e. exposure time, frame rate, etc) of the CCD camera optimized in order to maximize the detected signal without saturating pixels and to provide sufficient frames within each cycle of the driving oscillatory micropumps to allow confirmation that a steady signal is achieved and maintained, as described in earlier work [13]. An accompanying background image is also captured with the same acquisition settings as the aforementioned movie but under quiescent conditions. Subsequent image processing is performed using ImagePro Plus software (Media Cybernetics) and involves three steps: firstly the background image is subtracted from each frame of the movie to remove the excess background intensity; secondly each frame of the movie is divided by the background image. This second step represents an effort to correct for any variation in laser intensity over the field of view (e.g. the Gaussian profile of the laser spot). Finally, the intensity to retardation to birefringence transform is applied to the entire intensity-corrected movie.

As an example, Fig. 1a shows a false-colour image of a birefringent strand in the cross-slot device with a PSF solution at $20\times$ dilution ($f = 20$). The image is a single frame taken from a movie that has been processed as described above. The pumping frequency is 0.18 Hz, which in this case provides a strain rate of $\dot{\varepsilon} = 1346\ \mathrm{s}^{-1}$. The exposure time of the image is 20 ms and the acquisition rate is 33 fps (frames per second). Hence approximately 183 frames are captured during each pump cycle. In this particular case, the birefringence reached a steady state after approximately 10 to 15 frames had been acquired and Fig. 1a in fact shows the 27th frame of the movie.

It is clearly seen in Fig. 1a that the birefringent strand is highly localized about the stagnation point and the intensity decays rapidly within the horizontal exit channels (x-direction). A line profile across the birefringent strand, along the $x = 0$ axis, is shown in Fig. 1b. This is produced by averaging the pixel intensity between the vertical dashed lines centred on $x = 0$ that are superimposed in Fig. 1a. Fig. 1b shows clearly how the birefringence is peaked on the stagnation point streamline (i.e. the $y = 0$ axis). The peak value of the birefringence at $x = 0$, $y = 0$ (i.e. at the stagnation point), determined as described, is taken as the measurement value of Δn for each test fluid at each imposed strain rate; in this test case example for PSF solution at $20\times$ dilution and $\dot{\varepsilon} = 1346^{-1}$, $\Delta n = 0.87\times10^{-6}$.

It is vital for the best validity of the flow-induced birefringence measurements that the flow field remains stable, two-dimensional (i.e. nominally invariant through the channel depth) and symmetric. The development of flow instabilities in viscoelastic fluids in planar stagnation point flows in cross-slot devices has been reported and investigated by a number of authors in recent years [22–25]. In a very recent study employing a range of polymeric test fluids (including HA aqueous solutions) in planar stagnation point flow in an optimized-shape cross-slot device [26], Haward and McKinley [25] identified two distinct modes of instability depending on the fluid elasticity number, $El = Wi/Re$. For highly elastic fluids with $El > 1$, the flow became asymmetric about the stagnation point beyond a critical Weissenberg number, $Wi_{crit} > 3$. For low elasticity fluids with $El < 1$ an "inertio-elastic"

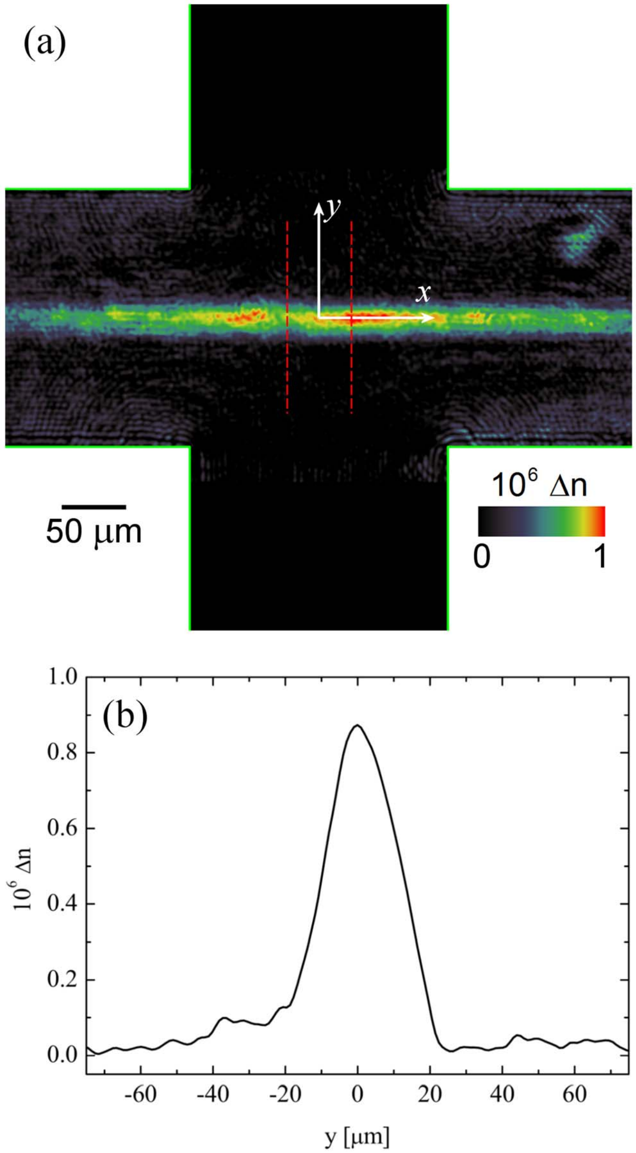

Figure 1. Birefringence in the cross-slots for a porcine synovial fluid (PSF) sample diluted by a factor of $20\times$ with phosphate-buffered saline (PBS). (a) An image of a birefringent strand at a nominal strain rate of $\dot{\varepsilon} = 1346\ \mathrm{s}^{-1}$. Flow enters through the vertical channels and exits through the horizontal channels. The image also indicates the coordinate system, with origin at the stagnation point. (b) A line profile across the birefringent strand where pixel values have been averaged between the dashed lines centred on $x = 0$ as shown in (a). The peak intensity at $y = 0$ can be taken as the measurement value of Δn for this test case.
doi:10.1371/journal.pone.0092867.g001

oscillatory mode of instability was reported beyond a (fluid dependent) critical Reynolds number, $Re_{crit} > 20$, or higher. In both cases, the onset of the flow instability can be clearly identified by the modified appearance of the birefringent strand – either a distortion of the strand about the stagnation point in the former case, or rapid spatio-temporal fluctuations of the intensity of the strand in the latter case (which most often appears as a smearing out or delocalization of the strand and a reduction in its intensity). Birefringence measurements made beyond the onset of instability in either case will be highly unreliable due to both the severely modified flow field and the high uncertainty associated with the

intensity of the strand. In the present experiments the elasticity number is quite low (El $\approx$ 0.5), so inertio-elastic flow instabilities would be expected to occur above some critical value of the Reynolds number. However flow instabilities are not the focus of the current investigation; here the main purpose is to investigate the conditions under which FIB measurements can be used to perform quantitative macromolecular characterizations on the HA in synovial fluid. Hence, all the reported measurements are made while the flow field remains stable, as is ascertained from the symmetry and uniformity of the birefringent strand in each case.

Results

3.1 Steady shear rheology

The results of steady shear experiments in the cone-and-plate rotational rheometer are presented in Fig. 2.

The least dilute fluid ($f = 10$) has a zero shear viscosity of around 2.4 mPas and is slightly shear thinning over shear rates $10\mathrm{s}^{-1} \leq \dot{\gamma} \leq 1000\mathrm{s}^{-1}$. The two more dilute fluids have similar viscosities of around 1.3 to 1.4 mPas that do not shear thin significantly over the range of shear rates tested. It is notable also that there is really a negligible difference between the behaviour of the diluted PSF and t-PSF samples. There is no evidence of the steep shear-thinning at low shear rates reported by Oates et al. [8,9] and Haward et al. [5] in model SF solutions and attributed by Oates et al. to structure-building by the protein content. However, this may not be surprising since in the works of Oates et al. and Haward et al. large differences between the rheology of HA solutions with and without proteins present were observed only at shear rates below $\dot{\gamma} \approx 1\mathrm{s}^{-1}$. For the dilute, low viscosity fluids under consideration here, the torque at such low shear rates falls below the noise floor of the rheometer, (indicated by the dotted line on Fig. 2).

3.2 Extensional flow birefringence measurements

Representative images of the birefringent strands observed in the cross-slots for two of the test fluids at strain rates spanning the range of measurement are presented in Fig. 3. Fig. 3a shows the birefringence observed in the PSF sample at 30× dilution, while Fig. 3b shows the birefringence observed in the t-PSF sample at 10× dilution. In both cases the birefringent strand remains symmetric and localized about the outflowing stagnation point streamline up to the highest applied strain rates, indicating the stability of the flow field at these rates, as discussed above. There is some background noise in the images, which is largely due to the presence of residual impurities in the fluids that unfortunately results in a less-than-perfect background correction. However, it is important to note that the intensity of the birefringent signal is easily measurable above the backbround noise, even in the most highly diluted PSF sample (i.e. Fig. 3a).

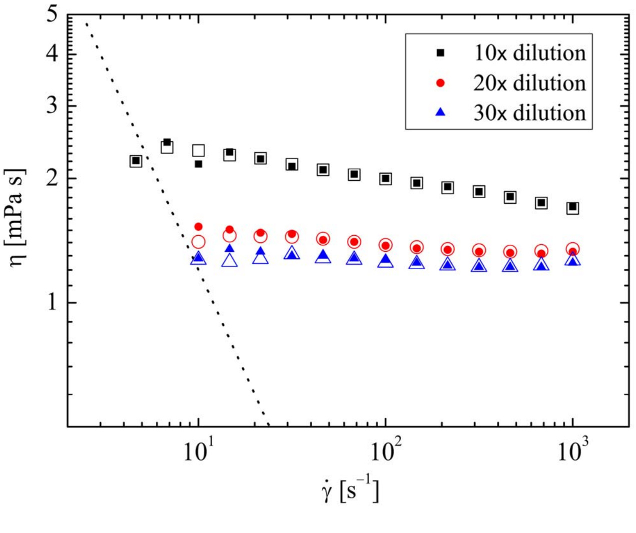

Figure 2. Flow curves of viscosity as a function of shear rate for the various test fluids composed of diluted porcine synovial fluid. Solid symbols: PSF, hollow symbols t-PSF. The dotted line represents the approximate sensitivity limit of the rheometer.
doi:10.1371/journal.pone.0092867.g002

Flow birefringence measurements made as a function of the strain rate at the stagnation point of the cross-slot device (as described in Section 2.5 and Fig. 1) are presented in Fig. 4. The results for the diluted PSF samples are shown in Fig. 4a, while Fig. 4b shows the results for the diluted t-PSF samples.

In all cases the birefringence becomes measureable at quite low strain rates of $\dot{\varepsilon} \approx 40\mathrm{s}^{-1}$, beyond which the signal increases monotonically up to eventual plateau values. The plateau birefringence $\Delta n_{\max}$ decreases as the dilution factor, f, increases and the concentration of HA in the fluid becomes reduced. The response of the diluted PSF and t-PSF solutions at each dilution appear to be very similar.

In Fig. 5 the value of the birefringence for all the fluids has been normalized by the dilution factor, f, and plotted again as a function of the strain rate. Here it is easy to see that there is indeed very little difference between the response of the PSF and the t-PSF fluids at each dilution factor. However, there is a systematic change in the fluid response as the dilution factor is increased; the birefringence tends to increase more rapidly as the strain rate is increased and to reach a greater eventual plateau value. These observations indicate that the fluids may not be truly dilute and that there may be some HA intermolecular interactions that are inhibiting the stretching of the HA macromolecules to some extent. Previous flow studies with solutions of a 4.8 MDa HA showed dilute behaviour for concentrations $c \leq 0.02$ wt.% [13], while light scattering data can be used to estimate the overlap concentration for such a molecule at $c^* \approx 0.04$ wt.% [27]. On the assumption that the HA concentration in the undiluted PSF is $c \approx 0.03$ wt.% [6], the concentration in the diluted samples would be around $c \approx 0.03$, 0.015 and 0.01 wt.% in the $f = 10$, 20 and 30 samples, respectively. It is very possible that the molecular weight of the HA in the assumption the plateau value of the birefringence ($\Delta n_{\max}$) can itself be used to estimate the HA concentration using the empirically-determined relation PSF sample is somewhat greater even than 4.8 MDa, which would shift the limit of dilute solution behaviour towards lower concentrations. In addition, the macromolecular stretching induced by strong extensional flows can lead to interactions between chain ends even when $c = c^*$ [28,29]. Therefore it is perhaps not surprising that there is some degree of residual HA intermolecular interactions in the test fluids. Even so, it seems reasonable to assume that at 30× dilution the PSF and t-PSF test fluids must certainly be approaching the dilute limit and on this $\Delta n_{\max} \approx 0.0084c$ [13]. Taking $\Delta n_{\max} = 0.65 \times 10^{-6}$ (see Fig. 4) a value of $c \approx 0.008$ wt.% is obtained. Multiplying this by the dilution factor of 30 yields an HA concentration in the pure PSF of $c \approx 0.24$ wt.%, which is in reasonable line with expectations for healthy fluid.

The birefringence versus strain rate curves can be used to extract macromolecular relaxation times. Point by point differences are used to differentiate the curves and they are plotted as a function of $1/\dot{\varepsilon}$ in Fig. 6. These curves represent the spectrum of longest relaxation times present due to polydispersity in the molecular weight distribution of the macromolecules in the fluid

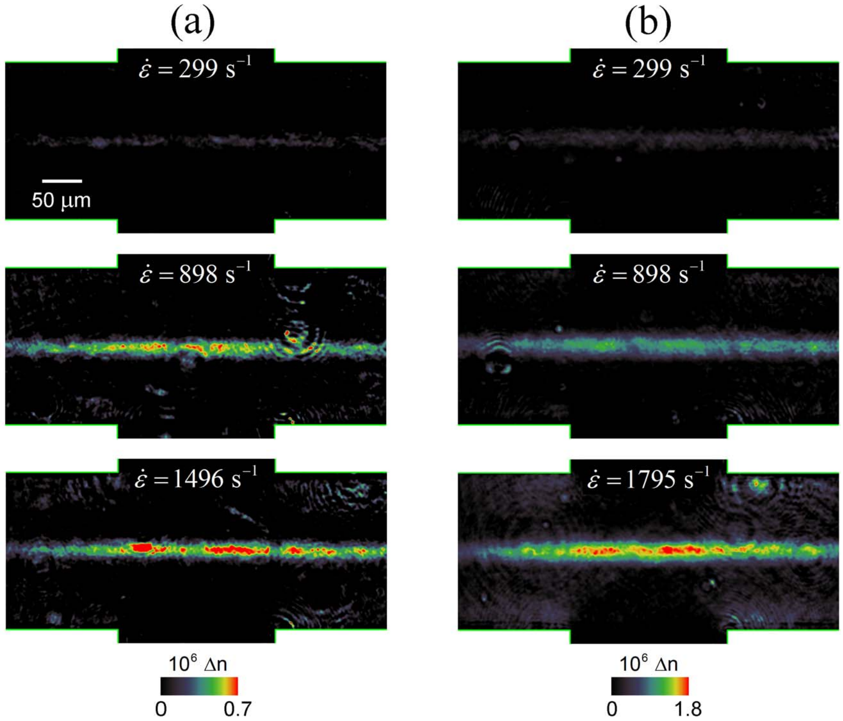

Figure 3. Images depicting the evolution of the flow-induced birefringent strand observed along the outflowing axis of the cross-slot device as the strain rate is increased for: (a) a PSF sample at 30× dilution, and (b) a t-PSF sample at 10× dilution.
doi:10.1371/journal.pone.0092867.g003

sample that contribute to the birefringence (i.e. predominantly the HA). They are well described by log-normal distribution functions, the peak of which can be used to determine a general characteristic relaxation time for the sample according to $\tau \approx 0.5/\dot{\varepsilon}$. Table 1 lists the relaxation times determined for all six fluids following this method.

A general reduction in the relaxation time is observed as the dilution factor is increased, consistent with the earlier observation that the fluids are tending towards dilution, but in which there is still some degree of interaction between HA chains. At lower dilution factors the relaxation time is longer due to interactions of the HA not only with the solvent but also with itself.

Using the relationship between relaxation time and molecular weight for hyaluronic acid, $\tau \sim M^{1.8}$ [13], the axes of Fig. 6 can be rescaled to yield the apparent molecular weight distributions of the HA present in the samples. The x-axes must be raised to the power of 1/1.8 (or 5/9) and scaled by an appropriate prefactor, which can be determined using a calibration standard of known molecular weight. The y-value of each point on the curve must by multiplied by a factor $\dot{\varepsilon}^{14/9}$, which serves to maintain the area beneath each fraction of the distribution curve. The resulting apparent molecular weight distributions are shown in Fig. 7. Table 2 lists the resulting values of the weight-averaged and number-averaged molecular weights (M_w and M_n) and the polydispersity index M_w/M_n.

It is seen in Fig. 7 that as the dilution factor is increased there is a small, but significant shift of the peak of the apparent distribution to higher molecular weight and also that the width of the distribution narrows. Of course, these effects are artefacts and not genuine, since the HA in each sample should be identical in all cases – the differences are again due to the changing degree of HA intermolecular interactions as the dilution increases and the corresponding effects on the macromolecular response to the extensional flow as revealed by the FIB measurements. Examination of Table 2 reveals that in fact there is no clear systematic effect on the weight-averaged molar mass, M_w, as the dilution factor is increased; the main effect is an apparent reduction in M_n. This causes a broadening in the apparent polydispersity index, M_w/M_n, and pushes the peak of the distribution towards lower molecular weight.

At the highest dilution ($f = 30$), where the analysis should be most accurate, there is really negligible difference between the derived molecular weight distributions of the PSF and t-PSF samples. Therefore the best estimate of the HA molecular weight, which results from these two samples, is $M_w \approx 5.5$ MDa with a narrow poydispersity index of $M_w/M_n \approx 1.3$. These parameters are quite consistent with those expected for the HA in healthy synovial fluid [6,30].

The lack of clear evidence for either the serum protein structure-building proposed by Oates et al. [8,9] or the lubricin-HA interactions proposed by Jay et al. [12] is perhaps a little surprising. Possible explanations for the absence of the former are more apparent: (a) possibly the effects reported by Oates et al. [8,9] were an artefact due to the air interface of the rheometer geometry, so are not observed here; (b) at the dilutions studied here any protein aggregates may be too small to form effective entanglements with the HA in the manner proposed by Oates et al.; or (c) if they were present, the protein aggregates were broken up in the extensional flow field. Such aggregates are only weakly associated with estimates of the interaction energy $\approx 3kT$ [31,32], and hence lifetimes of order microseconds, so can be easily broken up by flow-induced deformations. In the experiments of Oates et al. [8,9] the apparent protein structure-building was only reported at shear rates of $\dot{\gamma} \leq 1\,s^{-1}$, the explanation for this being that the protein structures could not sustain hydrodynamic forces at shear rates greater than this. In the present experiment, the minimum

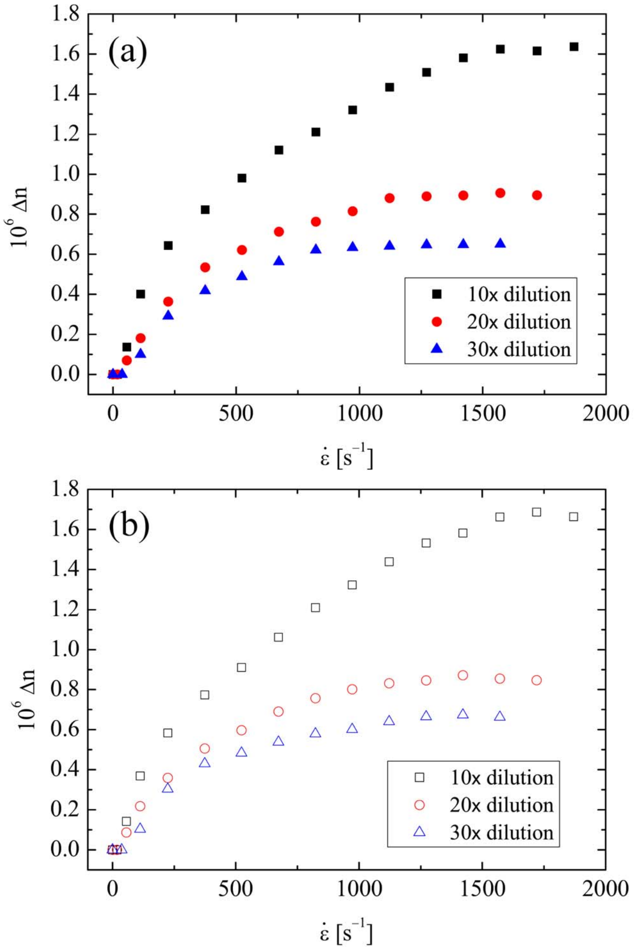

Figure 4. Birefringence as a function of the strain rate at the stagnation point of the cross-slots for (a) diluted PSF samples and (b) diluted t-PSF samples.
doi:10.1371/journal.pone.0092867.g004

extension rates probed are $\dot{\varepsilon} \approx 10\text{s}^{-1}$, corresponding to characteristic channel shear rates of the same order of magnitude. Even at the lowest strain rates at which the HA stretches, $\dot{\varepsilon} \approx 40\text{s}^{-1}$, it is likely that any serum protein aggregates (if they ever did exist) would have already been broken up before fluid elements had reached the stagnation point where the birefringence was being monitored. The likely total serum protein concentrations in the diluted PSF samples (c <0.2 wt.% and lower, depending on the dilution factor [7]) would have a minimal impact on the solvent viscosity if the proteins were not in an aggregated or structured state. In a microfluidic shear rheometer with no air interface, Sharma et al. [10] reported the viscosity of even a 1 wt.% solution of bovine serum albumin to have a viscosity virtually indistinguishable from that of the PBS solvent. Therefore the serum protein content in the diluted PSF samples is in all likelihood too small to have a measureable effect on the HA relaxation time through any modification of the solvent viscosity.

Explanations for the lack of any evidence for lubricin-HA interactions are less apparent. If, as suggested by Jay et al. [12], the lubricin causes a contraction of the HA equilibrium coil (by an unexplained mechanism) rendering it more flexible and deformable in flow, then this should result in a decrease in the HA relaxation time and should be readily detectable in the present experiment by a shift in the onset of birefringence to a higher

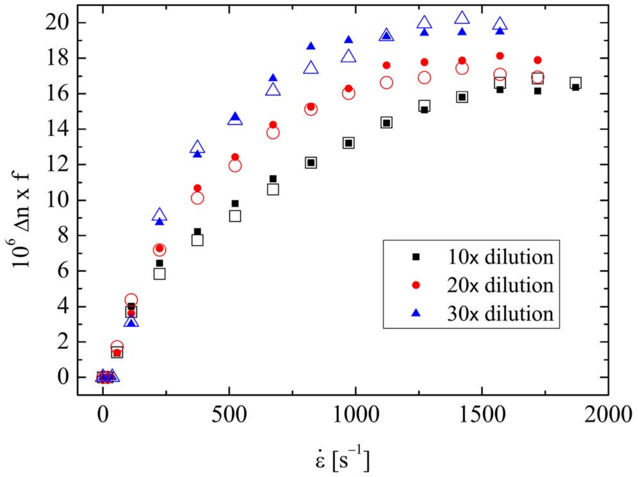

Figure 5. Birefringence normalized by the dilution factor for all the test fluids. Solid symbols: PSF, hollow symbols t-PSF.
doi:10.1371/journal.pone.0092867.g005

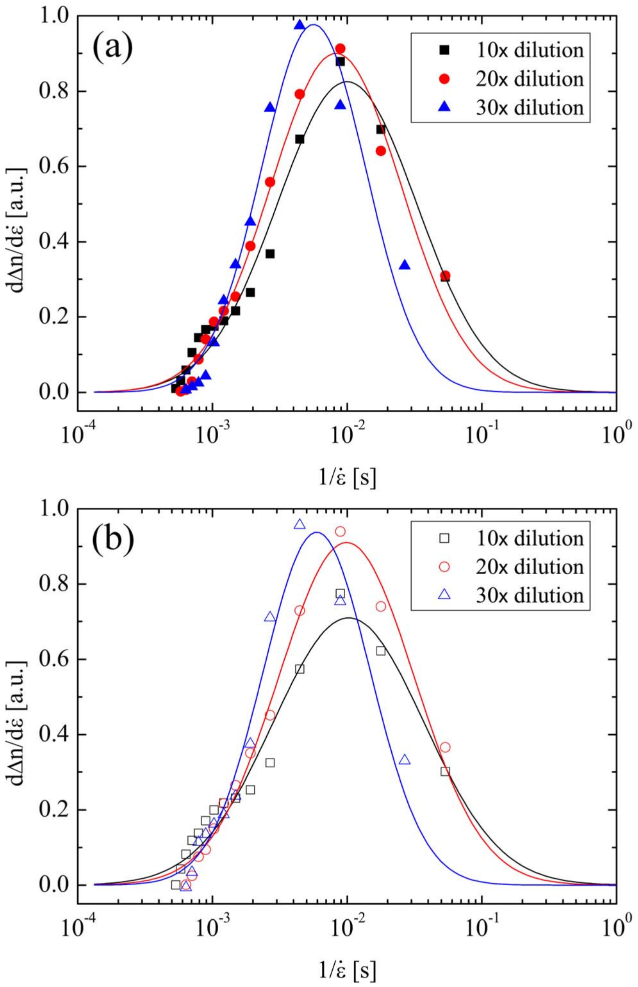

Figure 6. Relaxation spectra for (a) diluted PSF samples and (b) diluted t-PSF samples. The experimental data points (symbols) have been fitted with log-normal distribution functions (solid lines), the peaks of which can be used to obtain characteristic relaxation times for the HA macromolecules.
doi:10.1371/journal.pone.0092867.g006

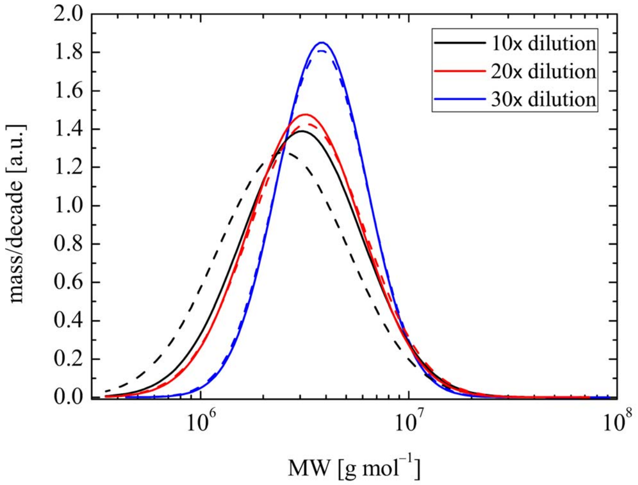

Figure 7. Apparent molecular weight distributions for PSF (solid lines) and t-PSF (dashed lines), derived from the log-normal distribution functions fitted to the experimental data shown in Fig. 5.
doi:10.1371/journal.pone.0092867.g007

strain rate. It may be that in the diluted PSF fluids tested here the lubricin concentration is simply too low have any measureable effect on the HA response. This would indicate the requirement of a critical minimum lubricin concentration for the interaction to be effective.

Conclusions

Birefringence measurements made as a function of the strain rate at the stagnation point of a microfluidic cross-slot device have been used to examine diluted solutions of porcine synovial fluid (PSF) and trypsin-treated porcine synovial fluid (t-PSF), in which the protein content had been digested by the trypsin enzyme. Despite the high sensitivity of the measurements to possible alterations in the macromolecular conformation, flexibility and relaxation time, at each fluid concentration tested no significant differences were observed in the evolution of the birefringence with strain rate between the PSF and t-PSF samples. Therefore in the present experiments, no conclusive evidence of protein-HA or protein-protein interactions in synovial fluid has been found. This does not necessarily discount the possibility that such interactions may play a role in the function of the synovial fluid in the joints, but simply means that in the diluted fluids tested here the absence of protein in the t-PSF samples had no appreciable effect on the fluid rheology or the macromolecular response to the extensional flow field in the cross-slots device. In the case of serum proteins, it is likely that even if protein aggregation did occur, the aggregates would have been destroyed at strain rates well below those at which birefringence due to orientation of the HA macromolecules occurred. In the case of lubricin-HA interactions, it is possible that the protein concentration must exceed a critical minimum threshold before such interactions begin to have noticeable effect on the HA conformation and relaxation behaviour.

Table 2. Weight-average and number-average molecular weights of the PSF and t-PSF samples as determined from analysis of the birefringence versus strain rate plots.

Fluid sample	M_w [10^6 g mol^{-1}]	M_n [10^6 g mol^{-1}]	*Mw*/*Mn*
PSF 30×	5.52	4.32	1.28
t-PSF 30×	5.60	4.32	1.30
PSF 20×	5.68	3.86	1.47
t-PSF 20×	6.05	4.00	1.51
PSF 10×	5.91	3.82	1.55
t-PSF 10×	5.41	3.23	1.67

doi:10.1371/journal.pone.0092867.t002

Far more significant than the presence or absence of protein in the test fluid is the concentration of the HA in the fluid. As the fluid is progressively diluted the birefringence develops more rapidly with strain rate and reaches a higher eventual plateau value relative to the concentration. This results in a reduction in the measured HA relaxation time and a narrowing of the derived apparent molecular weight distribution of the HA in the synovial fluid sample.

The results indicate that the digestion of protein is not a necessary step in the microfluidic synovial fluid HA analysis proposed in earlier work [13]. However the results of such an analysis can clearly be influenced somewhat by HA-HA intermolecular interactions, which must therefore be minimized in order for results to be most accurate. Hence it will be important to perform any such analysis at the highest dilution factor possible. The maximum dilution factor will only be limited by the sensitivity of the optics used for the FIB measurements and increasing the dilution factor will provide the significant advantages of reducing the quantity of SF required to perform each test and of diluting any impurities that remain suspended in the test fluid after centrifugation.

Next steps in the development of the research will therefore primarily focus on working towards maximizing the sensitivity of the FIB detection. It will also be important to test SF samples from a range of sources in order to gauge the variability in HA parameters between healthy donors as well as between donors from different species. This will eventually allow microfluidic screening of microscopic SF samples and the rapid assessment of whether or not the HA content of the SF lies within normal healthy parameter limits.

Author Contributions

Conceived and designed the experiments: SJH. Analyzed the data: SJH. Contributed reagents/materials/analysis tools: SJH. Wrote the paper: SJH.

References

1. Palfrey AJ, Davies DV (1968) Immediate viscosity of synovial fluid. J Appl Physiol 25: 672–678.
2. Al-Assaf S, Meadows J, Phillips GO, Williams PA (1996) The application of shear and extensional viscosity measurements to assess the potential of hylan in viscosupplementation. Biorheology 33: 319–332.
3. Bingöl AÖ, Lohmann D, Püschel K, Kulicke W-M (2010) Characterization and comparison of shear and extensional flow of sodium hyaluronate and human synovial fluid. Biorheology 47: 205–224.
4. Backus C, Carrington SP, Fisher LR, Odell JA, Rodrigues DA (2002) The roles of extensional and shear flows of synovial fluid and replacement systems in joint protection. In: Kennedy JF, Phillips GO, Williams PA, Hascall VC, editors. Hyaluronan Volume 1: Chemical, Biochemical and Biological Aspects. Cambridge, Woodhead Publishing Ltd. pp. 209–218.
5. Haward SJ, Jaishankar A, Oliveira MSN, Alves MA, McKinley GH (2013) Extensional flow of hyaluronic acid solutions in an optimized microfluidic cross-slot device. Biomicrofluidics 7: 044108.
6. Balasz EA, Denlinger JL (1993) Viscosupplementation: a new concept in the treatment of osteoarthritis. J Rheumatol Suppl 39: 3–9.

7. McCarty DJ (1997) Synovial fluid. In: McCarty DJ, Koopman WJ, editors. Arthritis and Allied Conditions. A Textbook of Rheumatology. Baltimore, Williams & Wilkins. pp. 81–99.
8. Oates KMN, Krause WE, Colby RH (2002) Using rheology to probe the mechanism of joint lubrication: polyelectrolyte/protein interactions in synovial fluid. Mat Res Soc Symp Proc 711: 53.
9. Oates KMN, Krause WE, Jones RL, Colby RH (2006) Rheopexy of synovial fluid and protein aggregation. J R Soc Interface 3: 167–174.
10. Sharma V, Jaishankar A, Wang Y-C, McKinley GH (2011) Rheology of globular proteins: apparent yield stress, high shear rate viscosity and interfacial viscoelasticity of bovine serum albumin solutions. Soft Matter 7: 5150–5160.
11. Elsaid KA, Jay GD, Warman ML, Rhee DK, Chichester CO (2005) Association of articular cartilage degradation and loss of boundary-lubricating ability of synovial fluid following injury and inflammatory arthritis. Arthritis Rheum 52: 1746–1755.
12. Jay GD, Torres JR, Warman ML, Laderer MC, Breuer KS (2007) The role of lubricin in the mechanical behaviour of synovial fluid. P Natl Acad Sci USA 104: 6194–6199.
13. Haward SJ (2014) Characterization of hyaluronic acid and synovial fluid in stagnation point elongational flow. Biopolymers 101: 287–305.
14. De Gennes PG (1974) Coil-stretch transition of dilute flexible polymers under ultrahigh velocity gradients. J Chem Phys 60: 5030–5042.
15. Larson RG, Magda JJ (1989) Coil-stretch transition in mixed shear and extensional flows of dilute polymer solutions. Macromolecules 22: 3004–3010.
16. Meyer F, Lohmann D, Kulicke W-M (2009) Determination of the viscoelastic behaviour of sodium hyaluronate in phosphate buffered saline with rheo-mechanical and rheo-optical methods. J Rheol 53: 799–818.
17. Keller A, Odell JA (1985) The extensibility of macromolecules in solution; a new focus for macromolecular science. Colloid Polym Sci 263: 181–201.
18. Haward SJ, Sharma V, Odell JA (2011) Extensional opto-rheometry with biofluids and ultra-dilute polymer solutions. Soft Matter 7: 9908–9921.
19. Haward SJ, Ober TJ, Oliveira MSN, Alves MA, McKinley GH (2012) Extensional rheology and elastic instabilities of a wormlike micellar solution in a microfluidic cross-slot device. Soft Matter 8: 536–555.
20. Odell JA, Carrington SP (2006) Extensional flow oscillatory rheometry. J Non-Newtonian Fluid Mech 137: 110–120.
21. Riddiford CL, Jerrard HG (1970) Limitations on the measurement of relaxation times using a pulsed Kerr effect method. J Phys D: Appl Phys 3: 1314–1321.
22. Arratia PE, Thomas CC, Diorio J, Gollub JP (2006) Elastic instabilities of polymer solutions in cross-channel flow. Phys Rev Lett 96: 144502.
23. Poole RJ, Alves MA, Oliveira PJ (2007) Purely elastic flow asymmetries. Phys Rev Lett 99: 164503.
24. Haward SJ, McKinley GH (2012) Stagnation point flow of wormlike micellar solutions in a microfluidic cross-slot device: Effects of surfactant concentration and ionic environment. Phys Rev E 85: 031502.
25. Haward SJ, McKinley GH (2013) Instabilities in stagnation point flows of polymer solutions. Phys Fluids 25: 083104.
26. Haward SJ, Oliveira MSN, Alves MA, McKinley GH (2012) Optimized cross-slot flow geometry for microfluidic extensional rheometry. Phys Rev Lett 109: 128301.
27. Mendichi R, Šoltés L, Schieroni AG (2003) Evaluation of Radius of gyration and intrinsic viscosity molar mass dependence and stiffness of hyaluronan. Biomacromolecules 4: 1805–1810.
28. Odell JA, Keller A, Müller AJ (1989) Extensional flow behaviour of macromolecules in solution. In: Glass JE, editor. Polymers in Aqueous Media. Washington, American Chemical Society. pp. 193–244.
29. Clasen C, Plog JP, Kulicke W-M, Owens M, Macosko C, et al. (2006) How dilute are dilute solutions in extensional flows? J Rheol 50: 849–881.
30. Fouissac E, Milas M, Rinaudo M (1993) Shea-rate, concentration, molecular weight, and temperature viscosity dependences of hyaluronate, a wormlike polyelectrolyte. Macromolecules 26: 6945–6951.
31. Gilson MK, Rashin A, Fine R, Honig B (1985) On the calculation of electrostatic interactions in proteins. J Mol Biol 184: 503–516.
32. Moon YU, Curtis RA, Anderson CO, Blanch HW, Prausnitz JM (2000) Protein–protein interactions in aqueous ammonium sulphate solutions. Lysozyme and bovine serum albumin (BSA) J Sol Chem 29: 699–718.
